# Editorial: The Social Life of Healthcare Decisions: Contexts and Consequences

**DOI:** 10.3389/fsoc.2022.839847

**Published:** 2022-03-15

**Authors:** Matthias Benzer, Rita Samiolo

**Affiliations:** ^1^Department of Sociological Studies, The University of Sheffield, Sheffield, United Kingdom; ^2^King's Business School, King's College London, London, United Kingdom

**Keywords:** healthcare, decision-making, sociology, evidence, social relations

The invitation to submit work to this Research Topic was issued with the aim of contributing to the sociological analysis of decisions about healthcare. It sought to encourage sociologists to engage with the social contexts in which such seemingly discrete decision-making “events” emerge and with their social consequences and ramifications.

The topic is consonant with several ongoing sociological concerns. For instance, sociologists have examined the changing notions of evidence and related forms of expertise that underpin decisions about healthcare (Timmermans and Kolker, 2004), highlighting key shortfalls of evidence-based medicine (Hanemaayer, 2020). Sociology has also examined how approaches to recommending which interventions be provided through publicly funded health systems may reshape social positions and relations (Benzer, 2020), while economics has grappled with the possibility that policies which increase average health may simultaneously intensify health inequalities in a population (Anand, 2002). The coronavirus pandemic has brought some of these issues into sharper focus and created the conditions for new controversies, for example controversies about decisions regarding which patients should receive life-prolonging care when demand exceeds resources (Campbell et al., 2020; Sayburn, 2020) or about what counts as informed consent (Corrigan, 2003) in respect of new types of vaccinations under emergency use authorization. By analyzing decisions as instances in which particular scientific rationales, social aspirations, political opportunities, and technological affordances temporarily coalesce, sociology can help make sense of the critical moments when what comes to be understood as “care” and “health” is being forged.

The articles for this Research Topic have tackled the topic as follows. Guided by the concept of pharmaceuticalisation and based on 20 interviews with General Practitioners (GPs) in England, Douglass and Calnan's paper discusses these doctors' understandings of and approaches to the preventative treatment of cardiovascular disease. In particular, Douglass and Calnan explore how different knowledges, treatment perspectives, and values shape the role of GPs in decision-making. They distinguish between doctors who endorse national guidelines and suggest practice should follow evidence-based medicine and doctors who are more skeptical of guidelines and critical of evidence-based medicine and who value professional experience. Moreover, they show that whereas some doctors favor recommending lifestyle changes alongside prescribing medication, others prefer the former to the latter, whilst yet another group emphasizes the benefits of medication. Finally, Douglass and Calnan spotlight judgements of specific components of a patient's individual circumstances and ethical evaluations that underlie considerations of what constitutes information relevant to patients. The paper situates GPs on a spectrum of pharmaceuticalisation regarding how they approach cardiovascular disease prevention.

Skyrme draws on her autoethnographic research in the United Kingdom to examine the decision to resort to a Buyers Club for acquiring health treatment at a comparatively low cost. She relates decision making to the experience of ill-health and the anticipation of health deterioration, the perception of—depending on the situation—time's passing too slowly or too quickly, and a sense of disconnection from much ordinary daily and public life. Moreover, Skyrme associates the decision with the awareness of the disease's infectiousness and notifiable status and of the stigma attached to it as well as with a feeling of social marginalization and isolation. Finally, she describes how the British National Health Service's policy to limit treatment provision to specific categories of patients and the welfare system's denial of social support in the form of financial benefits, which, she argues, was influenced by a narrow perspective on health and illness, have shaped the context of decision-making.

Schöngut-Grollmus et al. address the fiction of freedom of choice when healthcare options are restricted, as in the highly unequal Chilean context. Mobilizing the notions of dispositions and sociomedical networks, the authors follow chronically ill patients as they navigate the constraints of Chile's healthcare system during the disruption caused by Sars-Cov2 or as they confront the more generalized lack of care for patients with rare conditions. The authors document the financial, cultural, and medical ramifications of those chronic illnesses within a system ideologically wedded to freedom yet fraught with stark inequality. They question the notion of decision-making as a discrete and conscious act and show how feeling empowered to “make” a decision or rationalizing oneself as a decision-maker is a luxury reserved for the affluent.

Drawing on the theory of social representations, Lévesque and Negura examine how New Public Management reforms reshaped clinical social work in different Canadian cities. The authors illustrate how budget cuts, introduced within a context characterized by nominal professional autonomy, limited standardization of practice, and a humanistic professional ethos, have occasioned an array of coping strategies which make social workers operate in constant crisis management mode. Whilst social workers hold their professional autonomy dear, budget cuts have deprived them of the means to fulfill such autonomy. Consequently, social workers have begun to demand greater codification of their practice as a possible remedy for exploding workloads, lack of senior support, and heightened liability risk. Paradoxically, whilst reforms had not primarily targeted social workers' professional discretion, budget cuts created the conditions for a re-orientation of their professional self toward supporting greater standardization of practice, against the autonomy traditionally distinguishing the profession.

## Author Contributions

Both authors listed have made a substantial, direct, and intellectual contribution to the work and approved it for publication.

## Conflict of Interest

The authors declare that the research was conducted in the absence of any commercial or financial relationships that could be construed as a potential conflict of interest.

## Publisher's Note

All claims expressed in this article are solely those of the authors and do not necessarily represent those of their affiliated organizations, or those of the publisher, the editors and the reviewers. Any product that may be evaluated in this article, or claim that may be made by its manufacturer, is not guaranteed or endorsed by the publisher.
